# Supplementary material for: Dissecting the role of bHLH transcription factors in the potato spindle tuber viroid (PSTVd)-tomato pathosystem using network approaches
Source: PLoS One. 2025 May 7;20(5):e0318573. doi: 10.1371/journal.pone.0318573 (PMC12058033; doi:10.1371/journal.pone.0318573)
Supplement: Attachment — Submitted filename: Response to Reviewers.pdf [file pone.0318573.s003.pdf]

Re: Manuscript PONE-D-24-31516
Title: Dissecting the Role of bHLH Transcription Factors in the Potato Spindle Tuber Viroid-Tomato Pathosystem Using Network Approaches

Dear Editor and Reviewers,

We thank you for the thorough review and insightful comments on our manuscript *"Dissecting the Role of bHLH Transcription Factors in the Potato Spindle Tuber Viroid-Tomato Pathosystem Using Network Approaches"* submitted to PLOS ONE. We appreciate the opportunity to revise our work and have carefully considered each point raised during the review process. Below, we provide detailed responses to each comment from the reviewers and outline the revisions made to the manuscript to enhance its quality and clarity.

Reviewer #1: Comments and Responses

1. **Comment:** "Ten distinct gene co-expression modules, designated as M1 to M10, have varying sizes ranging from 216 to 23." Please clarify if the number of genes is decreasing across the modules.

   Response: We have clarified in the manuscript that the sizes of the modules do not follow a strictly decreasing order. The module sizes vary based on the number of genes they contain, which reflects the complexity and diversity of the co-expression patterns observed. This clarification has been added to the Results section *"In our analysis of root tissue, we identified ten distinct gene co-expression modules, labeled M1 to M10, with sizes ranging from 23 to 216 genes"*

2. **Comment:** "Module 2, comprising 123 genes, underscores the integral role of the bHLH transcriptional regulator, Solyc06g051550, in modulating iron uptake efficiency. This TF interacts with more than 400 genes in this module, highlighting its importance." The phrase "400 hundred" is incorrect.

   Response: The incorrect phrase "400 hundred" has been corrected to "*more than 400 genes*". We have thoroughly reviewed the document to ensure numerical accuracy.

3. **Comment:** "Two BHLH genes, Solyc06g051550 and SlbHLH036, act as central connectors." Please ensure consistent use of the term "bHLH" for

genes. Also, unify the naming of SlbHLH036 as Solyc05g006650.2 for consistency. The same is applied to bHLH083.

Response: We have revised the manuscript to consistently use the term “bHLH” for gene references. The naming of SlbHLH036 and bHLH083 gene symbols has been unified throughout the manuscript.

4. **Comment:** On Fig. 5A add a, b and c to determine interaction network and biological functions for M2, M4 and M5 respectively.

   Response: We have updated Fig. 5A to include labels A, B, and C, corresponding to the interaction networks and biological functions of modules M2, M4, and M5, respectively.

5. **Comment:** In the text where you mention Fig.8 (A-D) replace the capital letters with lowcase as it is depicted in the figure.

   Response: Response: The figure has been adjusted to use capital letters, consistent with the text where Fig. 8 (A-D) is referenced.

6. **Comment:** In the legend of Fig. 10 and inside the text replace capital letter in lowcase as it is depicted in the figure.

   Response: The format for figures requires the use of capital letters. Therefore, the legend of Fig. 10 and all related textual references have been adjusted accordingly. Additionally, all figures have been edited to follow this style.

Reviewer #2: Major Revisions and Responses

1. **Comment:** In Page3, the second paragraph, “Yet, the presence of unknown genes in plant genomes poses challenges in data interpretation” It is difficult to understand the transition here. How does co-expression network analysis interpretate the data related with unknown genes? This study seems not integrate the data of any unknown genes but the known bHLH gene family. Additionally, from “This network forms a graph of…..” to the end, the introduction of co-expression network seems repetition and boring.

Response: We have clarified the Introduction section, emphasizing that our study focuses on known bHLH genes and that co-expression network analysis can help hypothesize the unknown functions of not completely annotated genes through their associations within the network. This transition has been revised to improve clarity.

"These high-throughput approaches have significantly advanced our understanding of the global effects of viroid infections, uncovering extensive alterations in gene expression across various plant systems [10]. However, the presence of genes with unknown functions in plant genomes continues to pose a challenge for data interpretation. In our study, we focused specifically on known bHLH genes, employing co-expression network analysis to mitigate this challenge. By clustering genes based on shared expression profiles, this method enables the prediction of their functions through associations within the network, providing a powerful tool for functional annotation [20-22]"

Comment: How does co-expression network analysis interpretate the data related with unknown genes?

Response: Co-expression network analysis is a powerful tool for interpreting data related to unknown genes, especially when their functions are not well characterized. This method relies on the assumption that genes with similar expression patterns across various conditions or samples are likely to be functionally related or co-regulated. Here is how co-expression network analysis can help interpret data related to genes with unknown functions:

Identification of functional modules. Co-expression network analysis groups genes into clusters or modules based on their expression patterns. Unknown genes that cluster with well-characterized genes can be inferred to have similar functions or be involved in the same biological processes.

Gene function prediction: By analyzing the co-expression patterns, unknown genes can be assigned potential functions. For example, if an unannotated gene is consistently co-expressed with genes involved in stress response, it may play a role in that process.

Network centrality measures: Co-expression networks use metrics like degree centrality, or betweenness centrality to identify key genes known

as "hub" genes within a network. If an unannotated gene is a hub, it might be crucial in the regulation of the module, suggesting a significant role despite its unknown functional status.

Association with known pathways: Co-expression networks can be overlaid with pathway data. Unknown genes that are closely connected to genes in a specific pathway might be involved in that pathway, providing clues about their function.

"Co-expression network analysis groups genes into modules based on similar expression patterns, allowing the inference of functions for unknown genes clustered with well-characterized ones. By examining these patterns, potential functions for unannotated genes, such as involvement in stress response, can be predicted. Network centrality measures help identify "hub" genes, which may indicate key regulatory roles. Additionally, overlaying co-expression networks with pathway data can suggest unknown genes' involvement in specific biological pathways, providing further functional insights. Notably, genes with shared functions often exhibit robust correlations in their expression levels, laying the foundation for uncovering molecular pathways underlying diseases and conditions [31-33]."

2. **Comment:** In Page 4, the 4th paragraph, why only highlight "the tomato immune response" against viroid infection? Additionally, the sentence "we explore their involvement in host molecular mechanisms." seems incomplete.

   Response: We have broadened to highlight multiple pathways influenced by bHLH transcription factors, including immune responses as well as their roles in stress response, development, and signaling events during viroid infection.

   "*In this study, we aimed to identify co-expression modules of the bHLH transcription factor family, with a particular emphasis on their roles in diverse biological processes, including the tomato immune response to viroid infection. By positioning bHLH transcription factors (TFs) as hub genes, we explored their involvement in key host molecular mechanisms such as stress responses, development, and signaling pathways. Our primary objective was to uncover tissue- and strain-specific bHLH-guided transcriptional*

programs, highlighting both conserved and unique regulatory networks using network-based approaches"

Comment: Does bHLH play major roles in the resistance to viroid infection?

Response: Regarding the role of bHLH genes in viroid resistance, our study aimed to identify co-expression modules focusing on their functions in diverse biological processes. By positioning bHLH TFs as hub genes, we uncovered tissue- and strain-specific regulatory programs, emphasizing their roles in energy metabolism, membrane lipid repair in roots, and regulatory networks involved in leaf responses to PSTVd infection involved in the disruptions in cuticle function, shifts in metabolic processes, and alterations in biosynthesis pathways.

***"**Our results reveal that bHLH TFs are associated with regulation of genes involved in energy metabolism and membrane lipid repair functions in infected roots. Furthermore, our findings shed light on the intricate regulatory networks orchestrated by bHLH TFs in leaf tissue during PSTVd variant infections. The disruptions in cuticle function, shifts in metabolic processes, and alterations in biosynthesis pathways portray the plant's adaptive response to mitigate viroid impact"*

Comment: "we explore their involvement in host molecular mechanisms." seems incomplete.

Response: The sentence has been clarified to explain our exploration of these factors in host molecular mechanisms. "*By positioning bHLH transcription factors (TFs) as hub genes, we explored their involvement in key host molecular mechanisms such as stress responses, development, and signaling pathways"*

3. **Comment:** In Page 4, last paragraph, the first sentence, these is no solid molecular and genetic evidence that can prove that "bHLH TFs regulate photosynthesis and membrane lipid repair in infected roots". The co-expression network analysis only shows association not causal relationship.

Response: Thank you for your insightful comment. We acknowledge that co-expression network analysis identifies associations rather than establishing causality. Our statement aimed to highlight the potential roles of bHLH TFs based on their co-expression with genes involved in photosynthesis and membrane lipid repair in infected roots. We have revised the text to clarify that these findings suggest a possible regulatory role of bHLH TFs, supported by association rather than direct molecular or genetic evidence. Future experimental studies, such as gene knockouts or overexpression analyses, will be necessary to experimentally confirm these regulatory functions.

"Our results reveal that bHLH TFs are associated with regulation of genes involved in energy metabolism and membrane lipid repair functions in infected roots"

4. **Comment:** Page 6, the first paragraph, why chose "Microarray datasets"? Most of the gene in microarray analysis should be known, while RNA-seq analysis can reveal some unknown genes.

   Response: Thank you for your comment. While RNA-seq can identify novel genes, microarray data offer reliable insights, particularly for genes that are already annotated at the sequence level, as is the case with many genes in the tomato genome. Moreover, co-expression networks derived from microarray data enable functional inference for co-expressed genes, even when their annotations are incomplete, by leveraging their network associations. We selected the Corto algorithm, which requires a minimum of 20 samples to construct robust and reliable co-expression networks. This sample size criterion influenced our decision to use microarray datasets that met this requirement.

   "Despite the advantages of high-throughput data, we selected these datasets because they represent the most extensive resource currently available for studying gene expression in tomato under viroid infection. Furthermore, the sample sizes (n) for each dataset are sufficiently robust to generate reliable coexpression networks, ensuring the identification of key regulatory nodes and gene modules involved in tissue-specific responses."

Comment: Page 8, the second paragraph, the late sentence "This differential composition between the leaf and root……" This expression is

too conclusive. No strong evidence supports "the tissue-specific regulatory roles played by bHLH TFs,"

Response: The statement has been revised to suggest potential tissue-specific roles of bHLH TFs, noting that further experimental validation is required.

"The observed differential composition between the leaf and root networks suggests that bHLH TFs may have tissue-specific regulatory roles, potentially influencing distinct physiological, developmental, and metabolic processes in a tissue-dependent manner. However, further experimental validation is required to confirm these roles and to elucidate the underlying mechanisms"

5. **Comment:** Page 9, the first paragraph, generally, there is no chloroplast in root, how to explain the finding that "Genes with a single regulator are implicated in Chloroplast, Photosystems I and II,"?

   Response: We have clarified the reference to chloroplast-related genes and adjusted the text accordingly.

   "The finding that genes with a single regulator are enriched in chloroplast functions, such as Photosystems I and II, indicates a specialized regulatory mechanism. Although chloroplasts are primarily found in photosynthetic tissues like leaves, these genes may also be active in non-photosynthetic tissues like roots under certain conditions. For example, roots may activate chloroplast-related pathways in response to stress, such as viroid infection, which reflects an evolutionary remnant of gene regulation or cross-talk between metabolic and stress pathways"

   Discussion Section

   "Indeed, chloroplast genes, generally active in leaves, have been observed in roots during stress conditions, such as shoot removal. Genes like GOX1 and GLDP1 are upregulated in roots in response to cytokinin signaling, suggesting they contribute to redox regulation and metabolic adjustments in non-photosynthetic tissues (Feng et al., 2021; Kobayashi & Masuda, 2013). This behavior represents an "evolutionary remnant of gene regulation," where chloroplast-related genes, once solely for

photosynthesis, retain secondary functions and now aid plant stress responses across various tissues (Hayashi-Tsugane et al., 2014; Li et al., 2021)"

6. **Comment:** Page 11, the first sentence, "3.2.1 Modules that Exhibit Strong Symptom Induction in Response to the Severe Strain" What are the symptoms in root of tomato plants infected with viroid? and what associations of the following identified genes in this part with these symptoms?

   Response: We have added descriptions of root symptoms, such as stunted growth and root necrosis, and discussed how identified genes may be associated with these symptoms.

   "*Three modules, M2, M4, and M5, showed significant regulatory shifts during the 24-day symptom development phase of the severe PSTVd strain, as illustrated in Figure 5(A). These modules displayed a coordinated surge in gene expression Figure 5(B) as root symptoms—such as stunted growth, root necrosis, and reduced root mass—worsened (24 dpi). These synchronized expression patterns could suggest that these genes may play a role in the plant's stress response and the progression of these symptoms"*

7. **Comment:** Page14, "3.3. Exploring Leaf Tissue Gene Co-Expression Modules". The symptoms induced by viroid infection in tomato plants are obvious in leaf. Why did not analyze the module associated with symptoms in leaf like the analysis of root?

   Response: We conducted the same analysis for both root and leaf tissues. The approach involved identifying and exploring gene co-expression modules associated with observed symptoms in both tissues. By applying this consistent methodology, we ensured that the analysis of gene expression and the identification of key regulatory modules were directly comparable between roots and leaves, allowing us to gain understanding of the potential tissue-specific regulatory roles of the genes involved. We refer the reviewer to section "3.3. Exploring Leaf Tissue Gene Co-Expression Modules" where the analysis for the modules associated to leaf tissue are described.

8. **Comment:** Page 18, third paragraph, the sentence "This synchronization is an evolutionary response to aggressive pathogens." is difficult to understand. How to transmit to 'evolutionary response'? Additionally, "Notably, hub genes emerge as the linchpins, much like regulatory keystones, ensuring the efficiency of defense strategies." Author always wants to link identified genes or gene module with plant defense. However, these genes have many biological functions, not only related to plant defense. Thus, why only highlighted plant defense?

   Response: Thank you for your insightful comment. We have revised the text to clarify that the synchronization of gene expression may reflect an adaptive strategy, not solely focused on defense, but rather on the coordination of various biological processes that help the plant respond to multiple stressors, including but not limited to pathogen attack. These hub genes serve as key regulatory elements, integrating signals across diverse pathways that include metabolic adjustments, growth regulation, and structural integrity, in addition to defense mechanisms. For instance, Solyc06g051550 (heme-binding)
   and Solyc05g006650.2 (SlbHLH036) play critical roles not only in response to stress but also in maintaining cellular homeostasis and metabolic processes crucial for plant survival under challenging conditions. This broader perspective underscores their multifaceted regulatory influence, supporting the plant's capacity to manage a variety of internal and external cues.

   "Root tissue analysis identifies bHLH-guided regulatory processes such as heme-binding (M2), ubiquitin pathways (M4), and cell wall dynamics (M5) as central during symptom development under the severe strain. These biological events are synchronized with enhanced gene expression, suggesting the plant's complex adaptive response, which encompasses not only defense but also critical functions like stress tolerance, growth regulation, and metabolic balancing. Hub genes, including Solyc06g051550 (heme-binding) and Solyc05g006650.2 (SlbHLH036), act as central regulatory nodes. Their dense interconnectivity suggests they integrate signals from various pathways, acting as amplifiers of the plant's coordinated response to diverse stressors, not limited to pathogen defense but also playing roles in maintaining cellular homeostasis and structural integrity during stress conditions. This regulatory role highlights their

importance in balancing multiple physiological processes essential for plant resilience"

9. **Comment:** Many contents of "Discussion" are repetition of "Results". This part should focus one or several import point or problem, not mention each point relates to results. The most important point should be the discussion about the new findings or explanations about plant responses to viroid infection, especially compares with those findings obtained using transcriptome analysis, because the basic logic of this study is co-expression network analysis have advantages than transcriptome analysis and can further understand the data. However, the new findings or understandings about transcriptome data are not obvious in this manuscript.

Response: The Discussion section has been revised and improved it to reduce repetition focus on key findings, including new insights into bHLH TFs' roles in PSTVd infection and their broader implications.

Reviewer #2: Minor Revisions and Responses

1. **Comment:** In Page 3, the first paragraph, "Despite their tiny size of 246–401 nucleotides,". Now the size of the biggest viroid is 434 nt, which is much more than 401 nt.

   Response: We have corrected this to reflect the updated size range of viroids, including the largest known size of 434 nucleotides and updated the reference.

2. **Comment:** Last paragraph, "Analyzing modular gene co-expression within these networks unveils the systemic functionality of genes [23, 24-29]." Why not [23-29]?

   Response: The citation has been corrected.

3. **Comment:** Can't find the reference [10].

Response: References have been corrected and verified according to text editions.

4. **Comment:** "Emerging reports link viroid diseases to hormone pathways and transcription factor dynamics, disrupting plant gene expression landscapes [19]". The cited reference is not the most proper. Please check others.

   Response: We have replaced the cited reference with more appropriate sources that directly address the link between viroid diseases, hormone pathways, and transcription factors.

5. **Comment:** Page 11, 3.2.1.1., "400 hundred genes" means 40 thousand genes?

   Response: This was an error, and we have corrected it to "400 genes."

6. **Comment:** Page 19, the second paragraph can be moved to results.

   Response: The Discussion section has been revised and improved it.
