# Supplementary material for: Dissecting the role of bHLH transcription factors in the potato spindle tuber viroid (PSTVd)-tomato pathosystem using network approaches
Source: PLoS One. 2025 May 7;20(5):e0318573. doi: 10.1371/journal.pone.0318573 (PMC12058033; doi:10.1371/journal.pone.0318573)
Supplement: Attachment — Submitted filename: Response_to_Reviewers_auresp_2.pdf [file pone.0318573.s004.pdf]

Reviewer Report, PONE-D-24-31516R1

Thank you for assigning me the manuscript for review. It is a well-organized and a good piece of work that should be published, however I have few suggestions and few questions.

Response: Thank you very much for your valuable feedback and positive assessment of our manuscript. We appreciate your time and thoughtful suggestions, which we address below to further improve the manuscript.

Introduction Section:

1. Authors started with a phrase about viroids which is good but I suggest to add one line for their lack of protein expression and characteristics like this. Very briefly.

Response: Thank you for the suggestion. We have incorporated a brief sentence highlighting the lack of protein expression in viroids and their reliance on host enzymatic machinery as follows:

"*This lack of protein expression makes viroids unique among plant pathogens, relying entirely on host enzymatic machinery for replication and systemic movement"*

2. how widespread is PSTVd in major tomato-growing regions? Please mention this detail

Response: Thank you for this observation. We have added the following paragraph in order to detail the widespread presence of PSTVd in major tomato-growing regions, emphasizing its global distribution and the associated risks due to international trade and insufficient phytosanitary controls as follows:

"*PSTVd has been reported in major tomato-growing regions worldwide, including Europe, North and South America, Asia, and Africa, where it poses a significant threat to agricultural productivity (https://gd.eppo.int/taxon/PSTVD0/distribution; accessed on January 2025). Outbreaks in these regions are often associated with the international trade of infected plant material and inadequate phytosanitary controls, highlighting the importance of global surveillance and stringent management practices.*

3. why were tomato chosen as the model system for this study?

Response: Tomato (*Solanum lycopersicum*) was selected as the model system for this study due to its economic importance as a staple crop worldwide and its well-characterized genetic and transcriptomic resources. Additionally, tomato is a natural host of PSTVd, making it highly relevant for studying viroid-host interactions. The availability of robust genomic tools and its susceptibility to PSTVd infection provide an ideal framework for investigating the molecular mechanisms of viroid pathogenesis and its impact on plant physiology.

We have rephrase this paragraph in the introduction as follows:

"An illustrative instance can be observed in tomato (Solanum lycopersicum), a vital global agricultural commodity that yielded more than 130,812,947 million dollars in 2022 (http://faostat.fao.org/, accessed in May 2024). This staple crop faces a tangible threat from viroids, underscoring the potential impact of these infectious agents on essential food production systems.Tomato was selected as the model system for this study due to its economic importance as a staple crop worldwide and its well-characterized genetic and transcriptomic resources. This choice is particularly relevant given that Potato spindle tuber viroid (PSTVd), one of the most studied viroids, has a significant economic impact on solanaceous crops, including tomato."

Materials and Methods:

1. The mention of tools like *Corto*, *CEMiTool*, and *Mfinder* is clear. However, briefly explaining why these specific tools were chosen over alternatives could strengthen the methodology's justification.

Response: For the analysis of transcriptional regulatory networks, Corto was chosen over ARACNe due to its ability to accurately reconstruct networks with smaller sample sizes, a critical consideration given the dataset used in this study. To identify co-expression modules, CEMiTool was selected instead of WGCNA, as it provides an automated pipeline that simplifies the identification, visualization, and functional annotation of co-expression modules. For network motif analysis, Mfinder was employed for its specific capability to detect and analyze network motifs, offering deeper insights into key regulatory patterns, which are less accessible with general-purpose network analysis tools. To strengthen the justification for our methodological choices, we clarified the selection of Corto, CEMiTool, and MFinder as follows:

"*For the network analyses, we used Corto, CEMiTool, and MFinder due to their specialized capabilities. Corto was used to reconstruct tissue-specific transcriptional regulatory networks, emphasizing bHLH interactions (Steps (A)–(B)), leveraging prior biological knowledge for accurate inference. CEMiTool identified and enriched co-expression modules, providing system-wide insights into gene interactions (Step (C)). Bipartite networks for each tissue were then constructed using Python scripts derived from CEMiTool output files (Step (D)). Finally, MFinder detected network motifs within the co-expression networks, uncovering key regulatory patterns (Step (E)). These tools were chosen for their precision, ease of implementation, and alignment with the study's objectives."*

2. Mention the threshold used for Spearman correlation coefficients and explain how it was determined (e.g., significance level, biological relevance).

Response: Thank you for the suggestion; we have revised and rewritten this section as follows:

"The Corto algorithm, available on the CRAN repository of R packages, was used to infer gene regulatory networks (GRNs) from tissue-specific expression matrices [40].

For this analysis, we used an integrated expression matrix and the PlantTFDB tomato transcription factor list. Corto employs Spearman correlation to identify gene associations and applies the Data Processing Inequality (DPI) to eliminate indirect interactions, with bootstrapping to ensure robustness. The leaf network included 27 samples and 8080 features, while the root network had 26 samples and 8080 features. Spearman correlation thresholds of 0.837 (leaves) and 0.828 (roots) were applied, alongside a p-value threshold of 1×10^{-8} was applied to filter significant edges, and 100 bootstraps were performed to ensure robustness. From these global GRNs, subnetworks focusing on bHLH-TFs and their interactors were extracted, enabling detailed analysis of tissue-specific regulatory dynamics under PSTVd-induced stress"

3. The use of *Gprofiler* is appropriate, but details on how significance thresholds (e.g., FDR values) were determined would enhance reproducibility.

Response:We have incorporated the following sentence:

"…using an FDR threshold of < 0.05 to ensure statistically significant results…"

4. Were time points (17, 24, 49 dpi) treated independently, or were longitudinal patterns analyzed?

Response: Thank you for the question; we have clarified how time points were handled in each network type as follows:

"Time points (17, 24, and 49 dpi) were analyzed differently depending on the network type. In the global CEMiTool network, data from all-time points were integrated to create a comprehensive co-expression network. For module-specific networks, time points were treated separately to capture dynamic changes across stages, resulting in dynamic networks. Finally, in the bipartite networks, data from all-time points were combined again to provide an integrated overview of significant interactions"

Discussion section

1. how does the activity of bHLH TFs in roots contribute to stress adaptation versus their role in leaves in energy homeostasis or defense? Providing more explicit comparisons could clarify the versatility of these TFs in managing stress in different plant organs.

Response: Thank you for your insightful comments. We have expanded the discussion to provide a more explicit comparison of bHLH TF activity in roots and leaves. Specifically, we now highlight how bHLH TFs in roots contribute to stress adaptation by activating chloroplast-associated pathways for redox homeostasis and metabolic stability, while in leaves, these TFs prioritize energy reallocation and defense mechanisms, such as photosynthesis regulation and secondary metabolite biosynthesis. To address this, we inserted the following section:

The distinct activity of bHLH TFs in roots and leaves during PSTVd infection underscores their versatility in managing tissue-specific stress responses. Bipartite

network analysis in this study reveals complementary regulatory dynamics in these organs, highlighting their specialized roles in stress adaptation.

For instance in roots, GO analysis reveals the activation of chloroplast-associated pathways, including Photosystems I and II, despite the non-photosynthetic nature of this tissue. As previously discussed, this activation likely represents an evolutionary remnant of gene regulation or a form of metabolic crosstalk, allowing roots to leverage chloroplast-related pathways to maintain redox homeostasis and redirect energy metabolism toward survival mechanisms. Key regulators such as SlbHLH052 Fer involved in heme binding and iron homeostasis, illustrate the critical importance of structural resilience and metabolic adaptation in root stress responses to viroid infection.

In contrast, leaves exhibit a more complex regulatory network, involving 30 bHLH TFs and 1970 target genes. A unique feature of the leaf network is the presence of genes regulated by more than four bHLH TFs, highlighting the intricate coordination required to balance energy homeostasis and defense mechanisms. Leaf-specific bHLH TFs, such as GBOF-1 (Solyc06g072520.1) and SlbHLH022 (Solyc03g097820.1) prioritize metabolic regulation, and secondary metabolite biosynthesis to facilitate rapid responses to biotic stress.

The modular co-expression analysis reveals distinct tissue-specific dynamics in the plant's response to viroid infection, with root and leaf hub genes exhibiting entirely unique profiles. This specialization underscores the plant's systemic strategy to deploy tailored molecular mechanisms to address the specific environmental and metabolic challenges faced by each organ.

In roots, bHLH TFs integrate with jasmonic acid (JA) and ethylene signaling pathways to regulate nutrient uptake and maintain structural stability, ensuring effective stress adaptation. For example, Solyc06g034370.1 hub gene (Pectinesterase) modifies the cell wall, providing enhanced mechanical support and increasing root resilience during viroid infection. Additionally, ethylene-responsive genes, such as Solyc09g075420.2 (Ethylene-responsive transcription factor 2b), play a pivotal role in integrating ethylene signaling to further support redox balance and structural integrity.

In contrast, leaf-specific hub genes, such as Solyc06g073260.2 (NAD-dependent epimerase) and Solyc10g078300.1 (Single-stranded nucleic acid binding R3H protein), prioritize immune signaling and secondary metabolite biosynthesis. These processes effectively reallocate metabolic resources to strengthen pathogen resistance, aligning with salicylic acid (SA)-dependent systemic acquired resistance (SAR) and enhancing both localized and systemic defense mechanisms. The interplay of bHLH TFs with key hormonal pathways, including JA, ethylene, auxin and, SA, refines tissue-specific stress responses by coordinating distinct molecular and physiological processes.

In roots, modules associated with severe PSTVd infection at 24 dpi highlight key stress-adaptive pathways, including ubiquitin-mediated defense, and cell wall dynamics. These modules display temporal dynamics, with early induction and subsequent repression during recovery, particularly in genes related to photosynthesis, such as photosystem I reaction center subunit V (Solyc07g066150) and chlorophyll a-b binding protein 7 (Solyc10g006230). In leaves, most modules show repression, except for Module 2 (M2), which is linked to defense responses. Modules M1 and M3 Emphasize repression of chloroplast biogenesis and metabolic regulation, respectively, while Module 5 (M5), regulated by bHLH083 (Solyc03g118310), underscores the role of carbohydrate metabolism and energy homeostasis. Repression of chloroplast-related genes, including those involved in chloroplast biogenesis, reflects a strategic reallocation of resources from photosynthetic processes to defense, further emphasizing the prioritization of pathogen resistance in this tissue.

The differential regulation of chloroplast-related genes in roots and leaves highlights their distinct functional priorities during stress. In roots, the activation of chloroplast-associated pathways facilitates metabolic adjustments essential for stress tolerance, enabling redox homeostasis and energy redirection to survival mechanisms. In contrast, leaves repress these pathways to efficiently reallocate energy and resources toward defense, prioritizing the production of secondary metabolites and activation of immune responses. This divergence exemplifies a coordinated systemic response to viroid infection, with roots focusing on maintaining metabolic stability, while leaves concentrate on pathogen resistance and energy conservation. Moreover, these

processes, including chloroplast biogenesis and repair, not only sustain photosynthetic efficiency under stress but also drive the biosynthesis of secondary metabolites such as flavonoids and alkaloids, which are crucial for pathogen defense and overall plant resilience.

2. It is suggested to insert a sentence,

"The GO analysis identified several key categories, such as stress response, energy metabolism, and protein modification, which are significantly enriched in the identified gene modules, suggesting a broad functional role in managing PSTVd-induced stress."

Response: Thank you for the suggestion. We have adapted and incorporated the proposed sentence as follows:

"The GO analysis further supports this, identifying key functional categories such as stress response, energy metabolism, and protein modification, which are significantly enriched in the identified gene modules. This enrichment underscores the broad functional role of these modules in orchestrating the plant's systemic response to PSTVd-induced stress, integrating regulatory signals to optimize both defense and survival strategies."

3. *Please discuss the* chloroplast genes, as an evolutionary development

Response: Thank you for your suggestion. In the discussion, we have addressed the evolutionary significance of chloroplast gene regulation as follows:

"For instance, in roots, GO analysis reveals the activation of chloroplast-associated pathways, including Photosystems I and II, despite the non-photosynthetic nature of this tissue. This activation likely represents an evolutionary remnant of gene regulation or a form of metabolic crosstalk, allowing roots to leverage chloroplast-related pathways to maintain redox homeostasis and redirect energy metabolism toward survival mechanisms. Key regulators, such as SlbHLH052 Fer, involved in heme binding and iron homeostasis, illustrate the critical importance of structural resilience and metabolic adaptation in root stress responses to viroid infection."

Additionally, we highlighted the contrasting role of chloroplast genes in leaves:

"Repression of chloroplast-related genes, including those involved in chloroplast biogenesis, reflects a strategic reallocation of resources from photosynthetic processes to defense, further emphasizing the prioritization of pathogen resistance in this tissue. This divergence exemplifies a coordinated systemic response to viroid infection, with roots focusing on maintaining metabolic stability, while leaves concentrate on pathogen resistance and energy conservation."

These sections illustrate how chloroplast-related genes, originally dedicated to photosynthesis, may have evolved secondary roles in stress adaptation across

different tissues, reflecting an evolutionary and functional specialization in response to biotic stress.

4. While discussing the role of bHLH TFs, it might be useful to briefly mention how they interact with other transcription factors or signalling pathways involved in plant defence.

Response: Thank you for your suggestion. We have incorporated the following discussion on the interactions between bHLH TFs, targets and other transcription factors and signaling pathways involved in plant defense, emphasizing their integration of hormonal signaling to orchestrate systemic responses.

"In roots, bHLH TFs integrate with jasmonic acid (JA) and ethylene signaling pathways to regulate nutrient uptake and maintain structural stability, ensuring effective stress adaptation. For example, Solyc06g034370.1 hub gene (Pectinesterase) modifies the cell wall, providing enhanced mechanical support and increasing root resilience during viroid infection. Additionally, ethylene-responsive genes, such as Solyc09g075420.2 (Ethylene-responsive transcription factor 2b), play a pivotal role in integrating ethylene signaling to further support redox balance and structural integrity.

In contrast, leaf-specific hub genes, such as Solyc06g073260.2 (NAD-dependent epimerase) and Solyc10g078300.1 (Single-stranded nucleic acid binding R3H protein), prioritize immune signaling and secondary metabolite biosynthesis. These processes effectively reallocate metabolic resources to strengthen pathogen resistance, aligning with salicylic acid (SA)-dependent systemic acquired resistance (SAR) and enhancing both localized and systemic defense mechanisms. The interplay of bHLH TFs with key hormonal pathways, including JA, auxin, SA, refines tissue-specific stress responses by coordinating distinct molecular and physiological processes."
