# Supplementary material for: Dissecting the role of bHLH transcription factors in the potato spindle tuber viroid (PSTVd)-tomato pathosystem using network approaches
Source: PLoS One. 2025 May 7;20(5):e0318573. doi: 10.1371/journal.pone.0318573 (PMC12058033; doi:10.1371/journal.pone.0318573)
Supplement: Attachment — Submitted filename: Reviewer Report PONE-D-24-31516R1.docx [file pone.0318573.s002.docx]

**Reviewer Report,** PONE-D-24-31516R1

Thank you for assigning me the manuscript for review. It is a well-organized and a good piece of work that should be published, however I have few suggestions and few questions.

Introduction Section:

1. Authors started with a phrase about viroids which is good but I suggest to add one line for their lack of protein expression and characteristics like this. Very briefly.
2. how widespread is PSTVd in major tomato-growing regions? Please mention this detail
3. why were tomato chosen as the model system for this study?

Materials and Methods:

1. The mention of tools like Corto, CEMiTool, and Mfinder is clear. However, briefly explaining why these specific tools were chosen over alternatives could strengthen the methodology's justification.
2. Mention the threshold used for Spearman correlation coefficients and explain how it was determined (e.g., significance level, biological relevance).
3. The use of Gprofiler is appropriate, but details on how significance thresholds (e.g., FDR values) were determined would enhance reproducibility.
4. Were time points (17, 24, 49 dpi) treated independently, or were longitudinal patterns analyzed?

Discussion section

1. how does the activity of bHLH TFs in roots contribute to stress adaptation versus their role in leaves in energy homeostasis or defense? Providing more explicit comparisons could clarify the versatility of these TFs in managing stress in different plant organs.
2. It is suggested to insert a sentence,

"The GO analysis identified several key categories, such as stress response, energy metabolism, and protein modification, which are significantly enriched in the identified gene modules, suggesting a broad functional role in managing PSTVd-induced stress."

3. Please discuss the chloroplast genes, as an evolutionary development
4. While discussing the role of bHLH TFs, it might be useful to briefly mention how they interact with other transcription factors or signalling pathways involved in plant defence.
